# Reducing Behavioral Problems and Treatment Duration of Adolescents in Secure Residential Care: A Multiple Single-Case Experimental Design Study

**DOI:** 10.1177/01632787241228552

**Published:** 2024-12-02

**Authors:** Raymond V. Gutterswijk, Chris H. Z. Kuiper, Annemiek T. Harder, Frank C. P. van der Horst, Bruno R. Bocanegra, Peter Prinzie

**Affiliations:** 1 6984 Department of Psychology, Education & Child Studies, Erasmus University Rotterdam, Netherlands; 2 iHUB, Alliance of Youth Care, Mental Health Care and Special Educational Organizations, Netherlands

**Keywords:** adolescents, behavioral problems, secure residential youth care, repeated measurement, single case experimental design, treatment duration

## Abstract

Secure residential care (SRC) is criticized for several reasons. Therefore, in many countries, the general policy is to limit the length of stay of adolescents in SRC. However, research on length of stay and treatment effects of SRC on adolescents’ behavioral problems is sparse. Using single case experimental designs with time-series, forty adolescents referred to SRC completed a questionnaire on behavioral and attention problems every two weeks during a baseline (A) and treatment period (B). Two-level regression analyses were used to investigate the effects of SRC on behavioral and attention problems. In addition, we tested whether length of stay moderated effectiveness. On the individual level, the treatment showed a positive statistically significant effect on the behavioral problems of 0%–8% of the adolescents and a statistically significant negative effect on behavioral problems was found in 3%–10% of the adolescents. On the group level, adolescents showed no significant decrease in problem behavior or attention problems from baseline to discharge. Length of stay did not moderate the results. Based on the results we conclude that most adolescents fail to improve. In addition, length of stay was not associated with effectiveness, nor could it be explained by adolescents’ characteristics.

## Introduction

Several meta-analyses have reported that adolescents, who are referred to a secure residential youth care (SRC) facility because of their severe problem behavior, can benefit from treatment (De Swart et al., 2012; Knorth et al., 2007; Strijbosch et al., 2015). The most positive results regarding behavioral problems were found within SRC that was evidence-based (De Swart et al., 2012; Strijbosch et al., 2015), and in residential programs applying behavior-therapeutic approaches and stimulating family involvement (Knorth et al., 2007).

In several studies, researchers report the effectiveness of Dutch SRC, in addressing internalizing and externalizing problem behavior. Improvement in internalizing and externalizing problems is found in 22%–57% of adolescents, according to self-reports, from admission to discharge (Dirkse et al., 2018; Gevers et al., 2020; Gutterswijk et al., 2022). Regarding problems within the family, parents reported no improvement in family functioning during the treatment of their children in SRC. However, they did report a significant decrease in the parenting stress they experienced (Nijhof, 2011). Girls and boys tend to show similar behavioral and familial outcomes when leaving SRC (Gutterswijk et al., 2022). One of the fundamental elements to improve the effectiveness of these interventions is creating a positive living group climate, especially the safety experienced by the adolescents (Eltink, 2020).

However, SRC is the most expensive type of youth care that takes the youth out of their family environment (James, 2017; Knorth et al., 2007). Furthermore, SRC substantially restricts autonomy of the youth, one of the basic human needs (Ryan & Deci, 2017). Within SRC, professionals have the legal option to apply restrictive measures, including limiting (digital) social contact, conducting urine tests for narcotics use, and restricting the adolescents to their room. Since the United Nations (1989) states that every child should grow up in a family environment for the sake of the full development of his or her personality, an exception is necessary to make out-of-home care such as a SRC placement possible:

States Parties shall ensure that a child shall not be separated from his or her parents against their will, except when competent authorities subject to judicial review determine, in accordance with applicable law and procedures, that such separation is necessary for the best interests of the child. (United Nations, 1989, Article 9)

For this reason, it is general policy in many countries, including The Netherlands, to limit the length of stay of adolescents in residential care, including SRC as much as possible (Dutch Government, n.d.; Dozier et al., 2014; Zemach-Marom et al., 2012).

Studies on the relation between length of stay and treatment outcomes of (secure) residential care are rarely performed and their findings are mixed. Gevers et al. (2020) for example found no correlation between length of stay of adolescents in both open and secure residential youth care in The Netherlands and change in internalizing and externalizing behavior. However, we know that adolescents who make repeated use of SRC in the Netherlands, have undergone a significantly shorter first treatment period in SRC than adolescents who do not make repeated use of secure residential care (Koster et al., 2016). Another study showing more positive outcomes for adolescents with a longer stay was performed in Israel in educational residential care settings. The findings of this study showed adolescents with a longer stay to exhibit significantly fewer emotional and behavioral adjustment problems at discharge than the adolescents with a shorter stay in care (Hoffnung Assouline & Attar-Schwartz, 2020). Moreover, from family-style residential care we know that a longer lengths of stay are related to obtaining a high school education (Ringle et al., 2010) and that adolescents staying in care for over six months having better educational, employment, and criminality outcomes at 24 month follow-up, than adolescents staying in care for less than six months (Huefner et al., 2018). The authors also show how these outcomes are associated with significantly more positive long-term estimated financial societal benefit (Huefner et al., 2018).

In 2020 the average length of stay for adolescents in SRC in the Netherlands was 6.5 months (approximately 197 days) (Addink & Van der Veldt, 2022). The average length of stay for the adolescents in our study was 202 for Hestia and 2023 for Midgaard. Adolescents remain in SRC for as long as necessary and as short as possible. SRC is considered necessary when the adolescents safety is at risk, and there is a significant cocnern that the adolescent may discontinue the essential help they require. Unfortunately, it is not uncommon for adolescents to stay in SRC longer than deemed strictly necessary, as follow-up care (e.g., foster care or less restrictive residential care is not always readily available.

In an attempt to shorten the adolescents length of stay in SRC, Blankestein et al. (2021) developed the intervention ‘ThuisBest’ (‘HomeBest’); an intervention that combines SRC with multisystemic therapy (MST). The goal of this combination was to allow adolescents to return home after SRC more quickly. Their findings showed that treatment in SRC on average takes six months. However, when the SRC placement is combined with a strong evidence-based systemic intervention (i.e., multidimensional family therapy (MDFT), MST, relational family therapy (RFT)), this duration is reduced by six weeks, to an average treatment duration in SRC of 4.5 months. In contrast, when SRC is combined with a systemic intervention with a less strong evidence-base (i.e., attachment based family therapy (ABFT), flexible assertive community therapy (FACT), forensic ambulant systemic therapy (FAST), systemic therapy (ST)), the duration of SRC is on average 8.3 months. Given that some trajectories combining evidence-based systemic interventions and residential care are explicitly aimed at reducing the length of stay in residential care this finding is not entirely surprising (Rovers et al., 2019). Furthermore, a higher level of family-centered attitude of the sociotherapists also predicted a shorter length of stay of adolescents in residential care (Blankestein et al., 2021).

Although previous studies have linked length of stay to adolescent treatment effectiveness, little is known about treatment effect trajectories of adolescents during their stay in SRC. Previous studies are mostly limited to a longitudinal design with only measurements at admission and at discharge, and in some cases follow-up (e.g., Eltink et al., 2017; Gevers et al., 2020; Strijbosch et al., 2015). Designs with more than two (repeated) measurements are rarely applied. Furthermore, findings are usually presented at group level and the development of the individual is disregarded, which detracts from the fact that every adolescent may have a unique developmental trajectory. Consequently, it is not yet sufficiently known which length of stay is most appropriate and whether shortening or extending the stay of an individual adolescent may be beneficial. Moreover, it is also unknown why the treatment of some adolescents in SRC takes (much) longer than the treatment of others, knowledge that can be beneficial to shorten the stay of adolescents in SRC.

### The Present Study

In this study, we examine the developmental trajectories of adolescents from two secure residential youth care locations in The Netherlands. By using Single Case Experimental Designs (SCEDs) with biweekly measurements, the aim of this study is to gain knowledge on the individual behavioral development trajectories of adolescents during SRC. SCEDs allow the assessment of idiographic change processes (Tanious & Onghena, 2019). Furthermore, the study aims to determine whether the differing length of stay of adolescents can be explained by the seriousness of their problems at admission, the development of their problems during their stay, the family therapy they received, their gender and their destination after discharge. This knowledge can be used as a clinical tool in deciding whether to continue treatment after a certain period or to seek appropriate after-care services (Barnett et al., 2004). We will discuss the results in the light of the desire to minimize duration of residential youth care.

In this study, we address the following research questions:

(1) In what way do the total, internalizing and externalizing behavioral and attention problems of adolescent boys and girls in secure residential care develop over time, during treatment, and is this development moderated by the length of stay?
(2) Are there any differences between adolescents with a short stay (<6 months) and adolescents with a long stay (>6 months) in secure residential care regarding their behavioral (i.e., total, internalizing and externalizing), attention or family problems and age at admission, gender, destination after discharge, received family-oriented therapy and behavioral problems three months after discharge? The cutoff score of six months is chosen in line with other studies (e.g., Huefner et al., 2018).

#### Hypotheses

Regarding the first research question, we expect adolescents to show a gradual decrease of their behavioral and attention problems over time. Furthermore, since we assume that all adolescents who enter SRC suffer from severe behavioral problems and we expect all adolescents to experience a near-comparable level of problems at the time of discharge, we expect adolescents who stay in SRC for a relatively short period of time to show a quicker decrease of their problem behavior.

Regarding the second research question, since we expect all adolescents to experience a near-comparable level of problems at the time of discharge, we expect the behavioral, attention and family problems of adolescents with a relatively short stay to be less severe at admission, than the problems of adolescents with a relatively long stay in SRC. Furthermore, we expect that evidence-based family-oriented therapy associates with a shortened stay of adolescents in SRC. Lastly, we expect that adolescents with a longer stay transfer back home more often than adolescents with a short stay, since the transfer back home is expected to require more extensive preparation (Nijhof et al., 2012).

## Methods

### Participants

The study population consists of adolescents (aged 12–18 years) admitted to two secure residential youth care locations in The Netherlands (named Hestia; a girls-only facility and Midgaard; a mixed facility). In total, 91 adolescents admitted in 2018 and 2019 to one of the locations were asked by the first author to participate in this study. In both settings, the first six weeks of the treatment in SRC are seen as a stabilization and observation phase (=baseline phase). After being in SRC for six weeks, the actual treatment of the adolescents’ problems starts. Adolescents who left SRC within these six weeks (*N* = 18) were excluded from the study, since they did receive no or hardly any actual treatment. For these adolescents, SRC was, after the stabilization and observation phase, not considered the most appropriate option, for example because of psychiatric problems or a (mild) intellectual disability. In addition, the data of ten adolescents did not meet the criteria for usage in the analysis, since their ‘intervention’ period was too short to collect data on at least three timepoints. Furthermore, 23 adolescents did not agree to participate in the study, resulting in the participation of 40 (63%) adolescents (*M* age = 15.55 years, *SD* = 1.38 years, 56.0% girls). Problems within the family (i.e., insufficient parenting skills) were found in 32% of the participating adolescents, reported by their parents using the parenting stress questionnaire (OBVL, Veerman et al., 2014).

To determine whether the response group was representative for the eligible cases, we compared participants and non-participants on variables available for both groups. Participants and non-participants did not differ with regard to gender (44% vs 39% male), age (*M* = 15.55, *SD =* 1.38 vs *M* = 15.35, *SD* = 1.42), total behavioral problems (*M* = 6.75, *SD* = 5.08 vs *M* = 6.60, *SD* = 5.64), internalizing behavioral problems (*M* = 1.35, *SD* = 2.70 vs *M* = 1.30, *SD* = 1.70) and externalizing behavioral problems (*M* = 2.40, *SD* = 2.76 vs *M* = 1.50, *SD* = 1.18) or attention problems (*M* = 3.00, *SD* = 1.78 vs *M* = 3.80, *SD* = 4.21). However, participants stayed in residential care for a statistically significant shorter period of time (*M* = 208 days, *SD* = 121.71) than non-participants (*M* = 221 days, *SD* = 171.52); t (69) = −5.14. *p* < .05.

### Settings

Hestia is a facility offering treatment to vulnerable girls who are, for example, victims of commercial sexual exploitation. Within this gender-specific (‘girls-only’) facility, professionals use a trauma-sensitive approach. An important part of this trauma-sensitive approach is screening for PTSD-symptoms in all girls. Trauma therapy is offered in almost all cases. Midgaard offers “regular” help to boys and girls referred to SRC. The term “regular” is only used to indicate that the help at Midgaard is non-gender-specific help. Adolescents who show a (mild) intellectual disability or (serious) psychiatric problems that are so severe that treatment in SRC is not feasible, are excluded from treatment in Hestia or Midgaard. Adolescents referred to SRC receive treatment within a living group (24 hours/7 days) with a highly structured daily routine. The staff, led by a behavioral scientist, formulate a treatment plan within 6 weeks after admission. When problems within the family context were identified at admission, a family counselor is appointed to the family. In both setting sociotherapists try to achieve a positive living group climate to optimize treatment results (Van der Helm et al., 2018). Moreover, additional individual therapy (e.g., trauma therapy or family therapy) is offered when indicated by a behavioral scientist or psychiatrist. Lastly, for some of the adolescents, pharmacotherapy is used for the treatment of, for example, ADHD, depression or sleep problems.

In both settings, professionals use a solution-focused and system-oriented approach. Furthermore, a cognitive behavioral approach is used. Establishing a positive working climate with the adolescents, their parents and other persons from their social network is considered a key element in the treatment, including the use of shared decision making and informal mentoring.

### Procedure

Youth admitted to Midgaard between January 2018 and June 2018 and to Hestia between November 2018 and July 2019 were included in the study. We used two subsequent inclusion periods to be able to conduct the measurements. A researcher provided the adolescents with a questionnaire (on paper, in an attempt to maximize responses) every two weeks, and they were filled out by the adolescents during their entire stay in the residential care facility (*N* = 40) and three months after discharge (*N* = 30). Adolescents filled out the questionnaires ranging from seven to thirty-four times, with a mean of fifteen times. A written informed consent was obtained from the adolescents and their parents (or legal guardians) and the adolescents received a small compensation for their time in return. In addition, parents were presented questionnaires on behavioral problems and attention problems of their children and on problems within the family. These questionnaires (*N* = 48) were provided at admission only, at the facility or during a home-visit. When processing the data, the participants names were replaced by a code to pseudonymize the data, according to ethical guidelines, as tested for by the medical ethical review committee (TWOR – 2018-24).

### Measures

#### Case File Analysis

Information about the length of stay was calculated through the date of admission and the date of discharge, abstracted from the adolescents’ case files. The length of stay of adolescents ranged from 40–463 days. The age at admission of the adolescents, their gender, and whether they received family-oriented therapy were also extracted from their case files. Lastly, the destination after discharge of the adolescents was retrieved from their case files (i.e. going back home, going to live on their own or a follow-up intervention (e.g. residential care, foster care or family-style group care).

#### Brief Problem Monitor-Youth (BPM-Y) and Brief Problem Monitor-Parent (BPM-P)

The adolescents completed the Brief Problem Monitor-Youth (BPM-Y; Verhulst et al., 1997) every two weeks during their treatment. The BPM-Y questionnaire is the shortened version of the Youth Self-Report (YSR; Verhulst et al., 1997). Parents filled-out the Child Behavior Checklist (CBCL; Achenbach & Rescorla, 2001), which are part of the Achenbach System of Empirically Based Assessment (ASEBA) (Achenbach & Rescorla, 2001). Both questionnaires are used to identify psychosocial problems. The BPM-Y consists of 19 items, divided over three subscales, and one total scale. Answers are given on a three-point scale (0 = not true, 1 = sometimes true and 2 = very true). The subscales ‘internalizing behavioral problems’ (6 items, clinical cutoff score: ≥7) (e.g., ‘I feel worthless), ‘externalizing behavioral problems’ (7 items, clinical cutoff score: ≥7) (e.g., ‘I am disobedient at home’), and ‘attention problems’ (6 items, clinical cutoff score: ≥6) (e.g., ‘I don’t finish things I start on’) were used, as well as the ‘total behavioral problems’ (19 items, clinical cutoff score: ≥17) scale. The Cronbach alphas for the BPM-Y of both the internalizing problems scale and the externalizing problems scale was α = .94. The internal consistency of the attention problems scale was α = .74 and of the total scale α = .86.

To assess behavioral problems through parent-report the Dutch version of the CBCL (Achenbach & Rescorla, 2001; Verhulst & Van der Ende, 2013) was filled out by parents or substitute caregivers. Three subscales were used to asses internalizing (32 items) and externalizing behavioral problems (35 items), and attention problems (11 items). Answers are given on a three-point Likert scale (0 = *not true*, 1 = *sometimes true* and 2 = *very true*), (e.g., ‘My child argues a lot’). Scores between the 93^rd^–97^th^ percentile are considered ‘borderline’ and any score above the 97^th^ percentile is considered ‘clinical’ (Achenbach & Rescorla, 2001). Internal consistency of the internalizing problems scale and the externalizing problems scale in the present study was *α* = .90 and *α* = .94 respectively.

#### Parenting Stress Questionnaire (OBVL)

Family problems were assessed using the OBVL questionnaire. The OBVL is a self-administered questionnaire, measuring parenting stress (e.g., ‘I feel happy when my child is by my side’). In this study, we utilized the subscales ‘parent-child relationship’ (6 items) and ‘parenting problems’ (7 items). Respondents answer these questions using a 4-point-scale (1 = does not apply, 2 = applies a little, 3 = applies fairly and 4 = applies completely). Scores on the ‘problems in the parent-child relationship’ subscale range from 6 to 24, where a score of 14 or higher indicates severe problems, for which treatment is indicated. Scores on the ‘parenting problems’ subscale range from 7 to 28, where a score of 18 or higher indicates severe problems (Veerman et al., 2014). In the present study, the internal consistency for the ‘parent-child relationship’ subscale was α = .91, for the ‘parenting problems’ subscale α = .85, and for the total OBVL α = .92.

### Data Analysis

Missing scores occurred in almost all participants. In the baseline phase, 14 out of 40 (35%) adolescents had one or in some cases two missing values. During the intervention phase, 27 adolescents out of 40 (68%) showed missing values. Those adolescents showed an average of 16% missing values during the intervention phase. And lastly, at follow-up, 30 out of 40 adolescents completed the measurement, resulting in 25% missing values. Missing value analysis (Little’s MCAR test) revealed data were missing completely at random (MCAR) (*χ*^2^ (995) = 986,68; *p* = .568) (Little, 1988). To increase statistical power, missing data were imputed five times by using multiple imputation (Peng & Chen, 2018). Since data were missing in an arbitrary pattern, the Markov Chain Monte Carlo method was used (Schafer, 2002).

**Figure 1.**
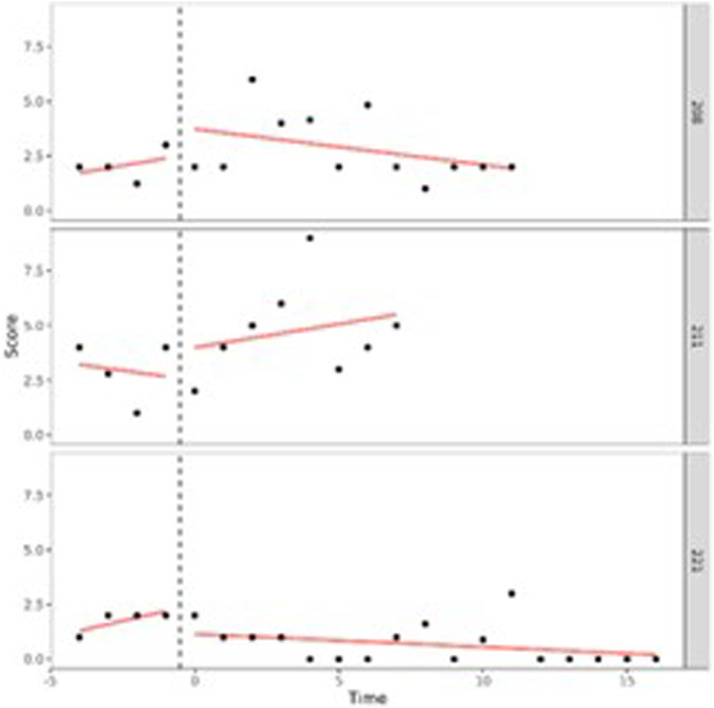
One-Level Regression Analyses of Total Behavioral Problems (*y*-Axis) Over Time (Two-Weekly) (x-Axis) After Centering the Time Variable – No Change Over Time.

**Figure 2.**
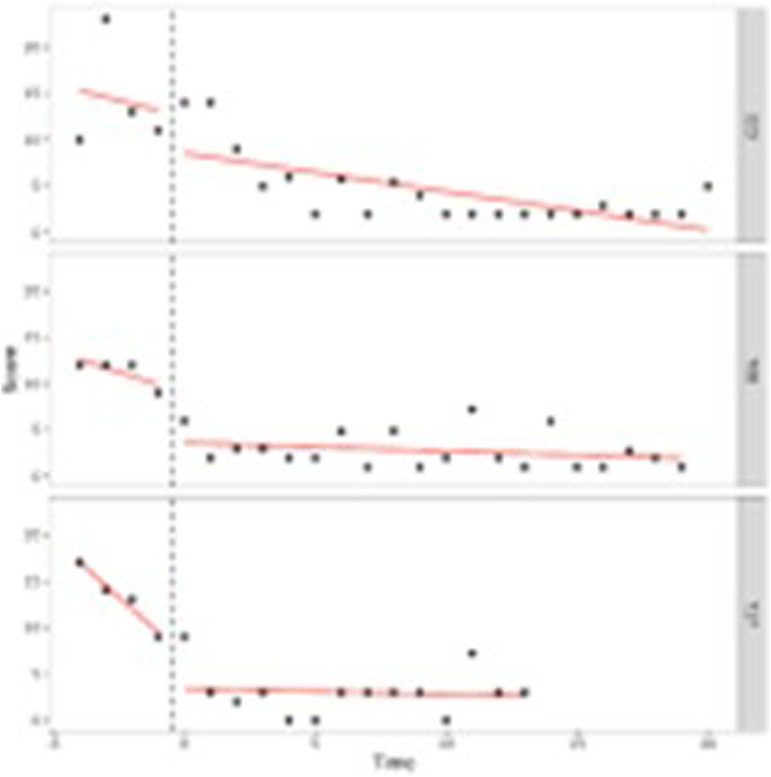
One-Level Regression Analyses of Externalizing Problem Behavior (*y*-Axis) Over Time (Two-Weekly) (x-Axis) After Centering the Time Variable – Decrease Over Time.

**Figure 3.**
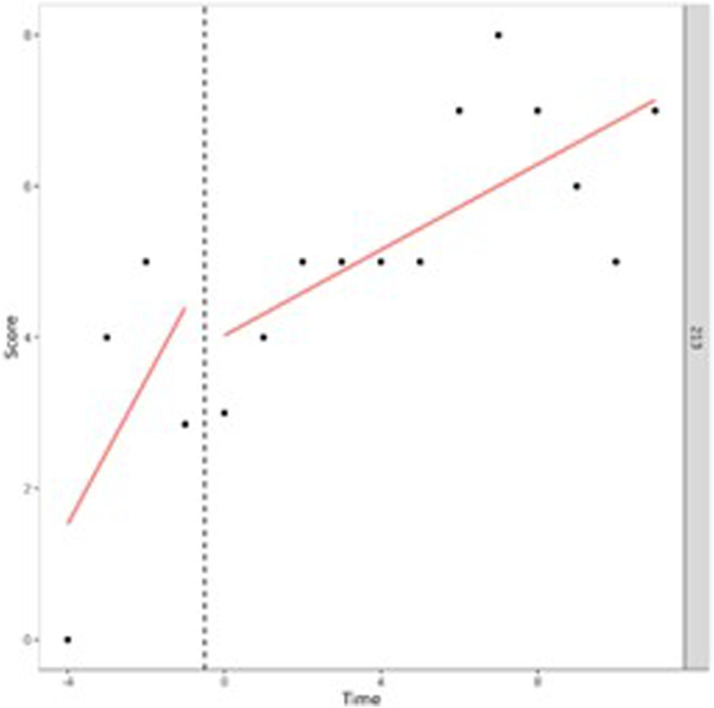
One-Level Regression Analysis of Externalizing Problem Behavior (*y*-Axis) Over Time (Two-Weekly) (x-Axis) After Centering the Time Variable – Increase Over Time.

**Figure 4.**
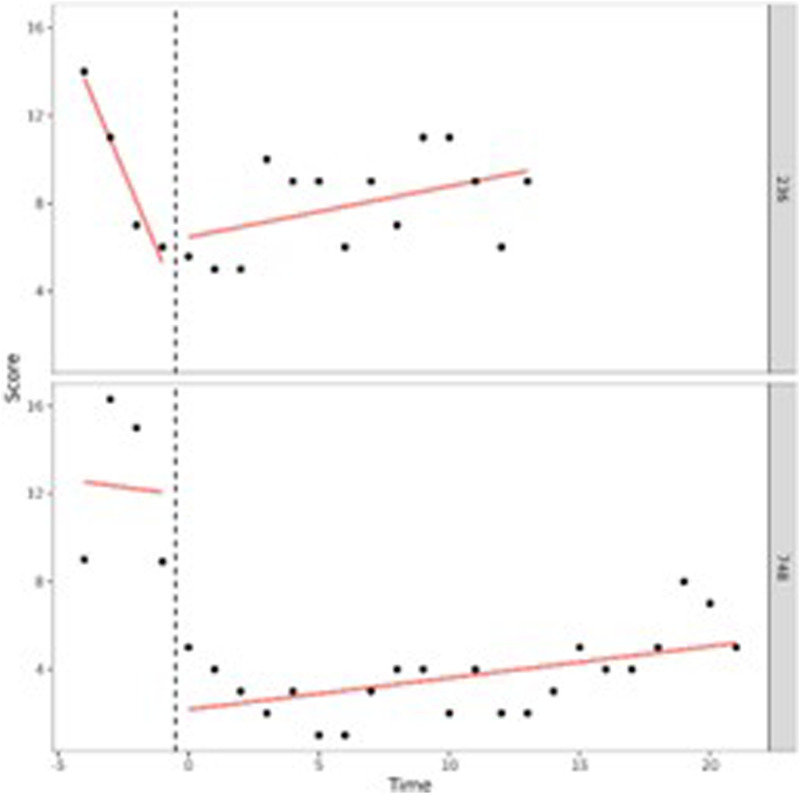
One-Level Regression Analyses of Total Problem Behavior (*y*-Axis) Over Time (Two-Weekly) (x-Axis) After Centering the Time Variable – Decrease and Increase Over Time (U-Shape).

Our first aim was to study in what way adolescents behavioral and attention problems developed during treatment. To do so, we performed (I) one-level (individual level) and (II) two-level (group level) regression analyses in MultiSCED (Declercq et al., 2020). As part of our first aim, the developmental trajectories of the adolescents were plotted in MultiSCED to be able to visually interpret in what way the behavioral and attention problems of the adolescents change over time (see Figures 1–4, for some examples). SCED is a rigorous research methodology with great suitability for the study of behavioral change. The most important added value of using SCED is the repeated, systematic assessment of an individuals’ dependent variables, improving internal validity, and enabling the execution of adequately powered statistical analyses, in order to detect fine-grained changes in outcomes, and tailoring interventions at the individual level (Bentley et al., 2019). SCEDs can reveal in what way behavior changes over time within adolescents, which is not well represented in findings from group-based studies. Furthermore, SCEDs allow to examine relationships between potential predictors of behavior over time and to explore individual response to an intervention (McDonald et al., 2017). The individual (one-level) developmental trajectories of the adolescents were plotted, according to the following equation:

$${\text{Score}}_{i}=\beta_{0}+\beta_{1}{Time}_{i}+\beta_{2}{\text{Phase}}_{i}+\beta_{3}{\left(\text{Phase}\times \text{Time}\right)}_{i}+e_{i}$$

With *β*_0_ being the intercept, which is the group mean score during the baseline period, *β*_1_ representing the increase or decrease during the baseline period, *β*_2_ representing the change of intercept after treatment starts and *β*_3_ representing the change of slope after treatment starts and the sampling errors (e_*i*_).

Two-level regression analyses were performed according to the following equation:

$${\text{Score}}_{ij}=\beta_{0}+\beta_{1}{Time}_{ij}+\beta_{2}{\text{Phase}}_{ij}+\beta_{3}{\left(\text{Phase}\times \text{Time}\right)}_{ij}+e_{ij}$$

$$\beta_{0j}=\gamma_{00}+\upsilon_{0j}$$

$$\beta_{1j}=\gamma_{10}+\upsilon_{1j}$$

$$\beta_{2j}=\gamma_{20}+\upsilon_{2j}$$

$$\beta_{3j}=\gamma_{30}+\upsilon_{3j}$$

In this equation, an index *j* is added to denote case *j* within the study. Each of the regression coefficients in the equation is divided into a fixed effect *γ* plus a random case-specific deviation *υ*, or random effect.

Lastly, to determine whether the level of behavioral and attention problems, and the effect of the treatment on the development of behavioral and attention problems, was moderated by the length of stay of the adolescent, the length of stay was added to the two-level regression analysis as a moderator, according to the following equation:

$${\text{Score}}_{ij}=\beta_{0}+\beta_{1}{Time}_{ij}+\beta_{2}{\text{Phase}}_{ij}+\beta_{3}{\left(\text{Phase}\times \text{Time}\right)}_{ij}+\beta_{4}{\text{Lengthofstay}}_{ij}+\beta_{5}{\left(\text{Lengthofstay}\times \text{Phase}\times \text{Time}\right)}_{ij}+e_{i}$$

$$\beta_{0j}=\gamma_{00}+\upsilon_{0j}$$

$$\beta_{1j}=\gamma_{10}+\upsilon_{1j}$$

$$\beta_{2j}=\gamma_{20}+\upsilon_{2j}$$

$$\beta_{3j}=\gamma_{30}+\upsilon_{3j}$$

In the equation above ‘length of stay’ is added as a moderator with *β*_4_ representing the moderating role of the length of stay on the level of behavioral problems during the baseline period and *β*_5 the_ moderating role of the length of stay on the change in slope after the treatment started.

Our second aim was to test for differences and similarities between adolescents who stay in SRC for a short period of time (<6 months) and adolescents who stay in SRC for a long period of time (>6 months). To do so, we used independent-samples *t*-tests for interval data. In the comparison of the behavioral, attention and family problems of the adolescents at admission, we used the first measurement after the adolescents had been admitted to SRC (i.e. within two weeks after admission). A Mann-Whitney *U* test was used when assumptions of the independent-samples *t*-test were violated (i.e., no normal distribution was found for self-reported internalizing (*W* = .83, *p* = .03), externalizing behavioral problems (*W* = .89, *p* < .01) and attention problems (*W* = .95, *p* = .03), and for parent-reported internalizing behavioral problems (*W* = .92, *p* < .01). To analyze the categorical data (i.e., destination after discharge, family therapy and gender), Chi-square tests were used.

As part of our second aim, differences in the behavioral and attention problems of adolescents at time of follow-up, between adolescents with a short stay and adolescents with a long stay, were explored. Only the last measurement of the adolescents’ behavioral and attention problems was used in these analyses (i.e., 3 months after discharge). Since one of the assumptions (i.e., normal distribution) of an independent-samples *t*-tests was violated, Mann-Whitney *U* tests were used. However, for the total behavioral problems the assumptions (i.e., independency of observations, no significant outliers, normality, and homogeneity of variances) for the use of an independent-samples *t*-test were met.

## Results

### Preliminary Analyses

Adolescents’ self-reported behavioral and attention problems at admission are shown in Table 1. Overall, adolescents report a relatively low level of total, internalizing, externalizing behavioral problems and attention problems at admission, with average scores, remarkably, below the clinical cut-off score.

**Table 1.** Behavioral and Attention Problems at Admission *(N* = 40).

Adolescents’ Problems	Clinical Cutoff Score	*M* (*SD*)	Clinical Problems *N* (*%*)
Total behavioral problems	≥17	9.22 (5.82)	4 (10%)
Internalizing behavioral problems	≥7	2.80 (3.08)	5 (13%)
Externalizing behavioral problems	≥7	2.84 (2.51)	5 (13%)
Attention problems	≥6	3.69 (2.47)	11 (28%)

### Development During Placement

#### Individual Development

A visual interpretation of the plotted developmental trajectories showed four types of developmental trajectories (see Appendix A, for more examples). The first type noticed showed the problems of the adolescents to be roughly stable over time (Figure 1), the second type showed the problems of the adolescents to (gradually) decrease over time (Figure 2), the third type showed problems to (gradually) increase over time (= negative development) (Figure 3), and the last type of development noticed showed the problems of the adolescents to decrease during the first weeks or even months of the treatment, however, after some time problems started to increase again (Figure 4). The plots of this last type of development consists of a ‘U-shape’.

**Figure 5.**
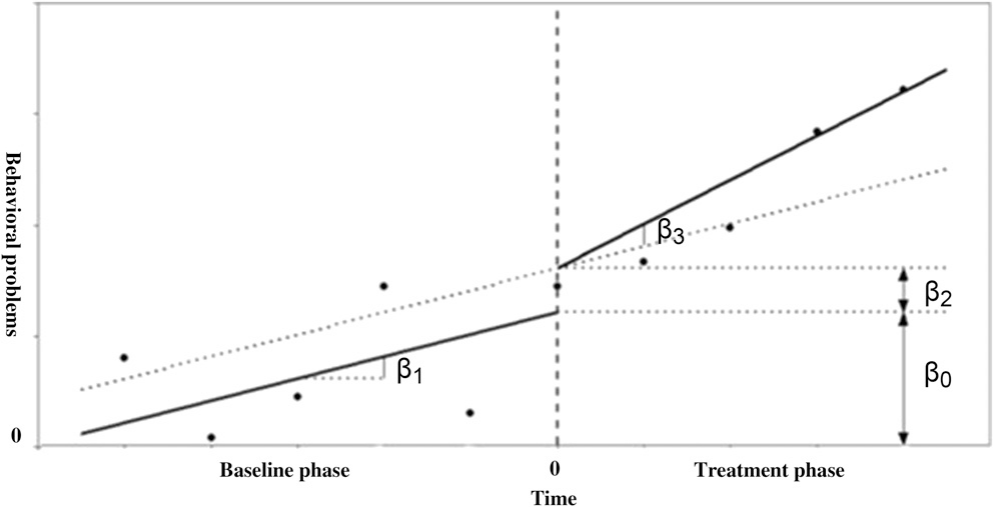
Graphical Interpretation of the Model Parameters After Centering the Time Variable. The Intercept is Expressed by *β*_0_, the Baseline Slope by *β*_1_, the Immediate Effect of the Treatment by *β*_2_ and the Effect of the Treatment on the Time Trend is Expressed by *β*_3_. *Note.* Reprinted from MultiSCED: A tool for (meta-)analyzing single-case experimental data with multilevel modeling, by Declerq et al. (2019), *Behavior Research Methods, 52,* p. 181.

The overall results of the analyses on the individual level are shown in Table 2. As can be seen from Figure 5 *β*_1_ represents the baseline slope (during the stabilization phase). Furthermore, *β*_2_ represents the immediate effect on the behavioral problems score of the start of the treatment. Lastly, *β*_3_ represents the effect of the treatment on the slope. For more details, see Appendix B.

**Table 2.** Adolescents Problem Development (*N* = 40) in Care.

Adolescents’ Problems		Baseline Slope (*β*_1_)	Immediate Effect of the Treatment (*β*_2_)	Effect of Treatment on the Slope (*β*_3_)
Total behavioral problems	Sign. decrease	5 (13%)	3 (8%)	3 (8%)
	Sign. increase	2 (5%)	3 (8%)	4 (10%)
	Non-sign. development	33 (82%)	36 (84%)	33 (82%)
Internalizing behavioral problems	Sign. decrease	4 (10%)	1 (3%)	1 (3%)
	Sign. increase	0 (0%)	2 (5%)	3 (8%)
	Non-sign. development	36 (90%)	37 (92%)	36 (89%)
Externalizing behavioral problems	Sign. decrease	3 (8%)	2 (5%)	0 (0%)
	Sign. increase	0 (0%)	2 (5%)	3 (8%)
	Non-sign. development	37 (92%)	36 (90%)	37 (92%)
Attention problems	Sign. decrease	2 (5%)	0 (0%)	2 (5%)
	Sign. increase	3 (8%)	1 (3%)	1 (3%)
	Non-sign. development	45 (87%)	39 (97%)	37 (92%)

From the findings in Table 2 it can be deduced that only a minor set of adolescents showed a statistically significant slope during baseline. Around 10% of the adolescents showed a statistically significant decrease in their problem behavior during the stabilization phase (*β*_1_). In addition, a minor set of adolescents experienced an immediate effect of the start of the treatment (*β*_2_). The direction of the effect differed between adolescents and showed to be both negative and positive. Depending of the type of problem behavior, 0%–8% of the adolescents experienced a positive treatment effect on the slope, and 3%–10% experienced a negative treatment effect on the slope, reflecting their problem behavior (*β*_3_).

#### Development on the Group Level

The analysis of the total behavioral problems, in MultiSCED, of the forty adolescents showed that, across cases, the total behavioral problems on average decrease statistically significantly by 0.75 [*t* (39.00) = −2.46, *p =* .02] per time unit (of two weeks) (*β*_1_) during the stabilization phase (baseline). During the stabilization phase, the outcome statistically significantly decreases to 8.70 points (*β*_0_) [*t* (45.87) = 4.43, *p ≤* .01]. The start of the treatment showed a statistically non-significant average immediate mean effect (*β*_2_) of 1.28 [*t* (44.47) = 0.72, *p =* .47]. The statistically marginal significant effect of the treatment on the slope (*β*_3_) was −0.10 [*t* (38.94) = 1.90, *p =* .06], indicating that the treatment decreases the time trend by −.10 points (−0.75 – 0.10 = −0.85).

Regarding internalizing problem behavior, the two-level analysis demonstrated that, across cases, the internalizing problem behavior statistically significantly decreases on average by 0.41 [*t* (39.00) = −3.02, *p* < .01] per time unit (of two weeks) (*β*_1_) during the stabilization phase (baseline). During the stabilization phase, the outcome statistically significantly decreases to 1.88 (*β*_0_) [*t* (41.42) = 2.41, *p* = .02]. The start of the treatment (*β*_*2*_) did not show a statistically significant effect: 0.27 [*t* (47.14) = 0.33, *p* = .74]. The statistically significant treatment effect (*β*_*3*_) on the slope was 0.34 [*t* (39.40) = 2.49, *p* = .02], indicating that the treatment increases the time trend by 0.34 points (−0.41 + 0.34 = −0.07).

For externalizing problem behavior, across cases, the problems statistically significantly decreases on average by 0.32 [*t* (39.00) = −3.04, *p* < .01] per time unit (of two weeks) (*β*_1_) during the stabilization phase (baseline). During the stabilization phase, the outcome statistically significantly decrease until 1.97 (*β*_0_) [*t* (40.88) = 2.47, *p* = .02]. The start of the treatment showed a statistically non-significant average immediate mean effect (*β*_2_) of 1.19 [*t* (42.12) = 1.65, *p* = .11]. The statistically significant treatment effect on the slope (*β*_3_) was 0.30 [*t* (39.03) = 2.84, *p* = <.01], indicating that the treatment increases the time trend by 0.30 points (−0.32 + 0.30 = −0.02).

Lastly, the development of the attention problems was analyzed. The two-level analysis in MultiSCED displayed that, across cases, the attention problems did not change statistically significant (*β*_1_) during the stabilization phase (baseline); 0.13 [*t* (39.00) = −1.00, *p* = .32]. At the end of the stabilization phase, the attention problems reached a score of 4.72 (*β*_0_) [*t* (46.31) = 5.04, *p* < .01]. The start of the treatment has no statistically significant immediate mean effect; of 0.51 [*t* (45.70) = 0.75, *p* = .45]. The analysis showed no statistically significant effect of the treatment on the slope: −0.06 [*t* (38.10) = −1.19, *p* = .24].

### The Moderating Role of the Length of Stay on the Treatment Effect on the Slope

Regarding total behavioral problems, the length of stay coefficient estimate indicates that for each week an adolescent stays longer in SRC, the average behavioral problems score is 0.10 points lower [*t* (36.97) = −0.97, *p* = .34], however, this effect of length of stay is not statistically significant. Furthermore, the estimates for the effect of length of stay on the treatment effect on the slope (length of stay × phase × time) is 0.002 for each extra week of stay in SRC [t (28.43) = 0.17, *p* = .87], but this finding again is not statistically significant.

In addition, the moderating role of length of stay on the level of internalizing behavioral problems was not statistically significant either, −0.01 [t (37.46) = −0.28, *p* = .78], neither was the moderating role of length of stay on the treatment effect on the slope, −0.001 [*t* (21.25) = −0.14, *p* = .89].

Regarding externalizing problem behavior, for every week the adolescents stayed in care longer, the problem score was 0.03 lower. However, this finding was statistically not significant [*t* (37.19) = −0.76, *p* = .45]. The moderating role of length of stay on the treatment effect on the externalizing problem behavior, again was not statistically significant, [*t* (14.51) = −0.17, *p* = .87].

Lastly, the length of stay did not statistically significant moderate the level of attention problems, −0.07 [*t* (36.23) = −1.56, *p* = .13], neither did length of stay moderate the treatment effect on the slope, 0.005 [*t* (24.56) = 0.87, *p* = .39].

### Comparison of Adolescents With a Short and Long Stay in SRC

In Tables 3 and 4 we present the results of the comparisons between adolescents leaving SRC within six months and adolescents staying in SRC more than six months. The results of this analyses were consistent with our analyses on the moderating role of the length of stay of adolescents in SRC.

**Table 3.** Comparisons Between Adolescents <6 Months in Care and Adolescents >6 Months in Care.

	Adolescents <6 Months in Care			Adolescents> 6 Months in Care			t	U
	*N*	M	SD	*N*	M	SD		
Behavioral problems								
Total behavioral problems^a^^,b^	29	9.31	6.91	21	9.86	4.10	−0.32^ns^	
Externalizing behavioral problems^a,c^	29	2.86	2.68	21	2.90	3.08		−.63^ns^
Internalizing behavioral problems^a,c^	2^b^9	2.76	3.18	21	3.05	2.38		−.15^ns^
Attention problems^a,c^	29	3.69	2.66	21	3.90	2.07		−.66^ns^
Total behavioral problems^b,d^	28	74.54	28.59	18	68.61	23.45	.73^ns^	
Externalizing behavioral problems^b,d^	28	30.71	12.96	19	28.95	12.04	.46^ns^	
Internalizing behavioral problems^c,d^	28	16.54	7.32	19	17.47	9.70		−.03^ns^
Attention problems^b,d^	28	7.29	4.16	19	7.42	5.34	.36^ns^	
Family context								
Low quality parent-child relationship^b,d^	29	11.93	5.33	19	12.16	5.18	−0.15^ns^	
Parenting problems^b,d^	29	17.86	4.77	19	16.95	4.70	0.65^ns^	
**Age**^b^	**29**	**15.22**	**1.18**	**21**	**16.01**	**1.53**	−2.07*	

*Note.* Significant differences are presented in bold.

*p < .05.

^a^Adolescent-report.

^b^Independent-samples *t* test.

^c^Mann-Whitney *U* test.

^d^Parent-report.

**Table 4.** Comparisons Between Adolescents <6 Months in Care and Adolescents >6 Months in Care.

	Adolescents <6 Months in Care		Adolescents >6 Months in Care		Test
	*N*	*%*	*N*	*%*	
Destination after discharge					*χ*^2^ (2,*N* = 50*) =* .60^ns^
Back home	11	38	10	48	
Follow-up intervention	17	59	10	48	
Living on their own	1	3	1	4	
Family therapy					*χ*^2^ (1,*N* = 50*) =* .11^ns^
Yes	18	62	14	67	
No	11	38	7	33	
***Gender***					***χ*** ^**2**^ **(1,*N* = 50*) =* .01** ^*^
Boy	17	59	5	24	
Girl	12	41	16	72	

*Note.* Significant differences are presented in bold.

*p < .05.

Power analyses revealed that the sample size was large enough for the t-tests and Mann-Whitney U tests to detect a large effect. Adolescents remaining in care for a longer period are slightly older at the time of admission; *t* (48) = −0.31, *p* < .05, and more often female, χ^2^ (1) = 5.99, *p* < .05.

### Behavioral and Attention Problems at Follow-Up

As can be seen in Table 5, the comparison of the problems of adolescents with a relatively short stay (<6 months) and adolescents with a relatively long stay (>6 months) did not differ statistically significantly for total, externalizing and internalizing behavioral problems at follow-up. Nor did the attention problems of adolescents staying for a short period of time differ from the attention problems of adolescents staying in care for a longer period.

**Table 5.** Follow-Up Scores of Adolescents With a Short and a Long Stay in SRC.

	Short Stay (<6 Months)			Long stay (<6 months)			*t*	*U*
	*N*	*M*	*SD*	*N*	*M*	*SD*		
Total behavioral problems^a,b^	20	6.75	5.08	10	6.60	5.64	.07^ns^	
Internalizing behavioral problems^a,c^	20	1.35	2.70	10	1.30	1.70		−.47^ns^
Externalizing behavioral problems^a,c^	20	2.40	2.76	10	1.50	1.18		−.67^ns^
Attention problems^a,c^	20	3.00	1.78	10	3.80	4.21		−.16^ns^

^a^Self-report.

^b^Independent-samples *t* test.

^c^Mann-Whitney *U* test.

## Discussion

The first aim of this study was to determine in what way the emotional, behavioral and attentional problems of adolescents develop during their stay in SRC. We expected adolescents to show a gradual decrease in their problems while offered intensive treatment. Contrary to our expectations, on average on the group level, adolescents’ emotional, behavioral and attention problems fail to decrease during their placement in SRC. However, the analyses on the individual level showed the development of adolescents’ problems varied widely between individuals. On the individual level, total, internalizing and externalizing behavioral problems and attention problems decrease in respectively 13%, 10%, 8% and 5% of the adolescents during the stabilization phase. The start of the treatment shows an immediate positive effect on the behavioral problems in 0%–8% of the adolescents, and in 0%–8% of cases a positive effect of the treatment was found on the slope. These findings show that, depending on the type of problems, at least 92% of adolescents fail to improve during treatment, compared to the stabilization phase. This means that despite the drastic out-of-home placement, the intensive treatment and the relatively high costs, only few adolescents seem to profit from treatment in SRC. A possible explanation for adolescents failing to improve is that for them, treatment in SRC is not suitable, given that these adolescents show non-clinical problems at admission. After all, previous research has shown, especially adolescents with the most severe problem behavior benefit from treatment in SRC (Gutterswijk et al., 2022; Nijhof et al., 2011).

In our sample, remarkably, a significant number of adolescents report no (behavioral) problems at admission, while placement in SRC should only be an option when adolescents cannot be treated at home or through less restrictive care. Since problems within the familial context are present for a third of the adolescents at admission, this may indicate that placement in SRC is, for some adolescents, the outcome of problems within the family and poor parenting by their parents. However, the general policy is to offer foster care or residential care first tot adolescents who cannot receive treatment at home. We assume for these adolescents without behavioral problems at admission, no suitable, less restrictive, care was available (Nijhof et al., 2011; Van Dam et al., 2010). Furthermore, a possible explanation for self-reports of adolescents not showing significant problem behaviors is limited problem awareness or problem recognition among adolescents. The fact that some adolescents (around 10%) already showed a decrease in their behavioral problems during baseline (stabilization phase) may have reduced the effect of the treatment in the analyses. Moreover, lack of evidence of a treatment effect is not necessarily evidence for the absence of a treatment effect. Our findings using SCEDs showed much smaller success rates on the individual level, than the results using the reliable change index (RCI). In a previous study using the RCI success rates of 23%–57% were found in improving (internalizing and externalizing) behavioral problems, according to self-reports, from admission to discharge (Gutterswijk et al., 2022).

Another possibility for adolescents failing to show improvement is prompted by the possible iatrogenic effects of SRC critics warn about. In our study, deterioration of behavioral problems during the stabilization phase was seen in 0%–8% of adolescents (see Table 2) and a negative effect of the treatment was found in 3%–10% of adolescents. In addition, adolescents failing to report problems at admission can possibly be explained by adolescents’ lacking problem awareness or pretending to behave better than they do by adapting behaviors to the SRC treatment environment (Harder, 2018). In addition, previous research in Dutch SRC showed parents to report behavioral problems of their children to be more severe than adolescents themselves do (Gutterswijk et al., 2022). In comparison to parent reports, adolescent report fewer behavioral problems at admission. According to self-reports, clinical problems are found in 10% of adolescents regarding total behavioral problems, in 13% of adolescents regarding both internalizing and externalizing behavioral problems and in 28% of adolescents regarding attention problems. In parent reports, higher percentages of respectively 63%, 81%, 74% and 83% are found. These differences might be explained by the fact that adolescents view their problems differently than their parents (or professionals) and experience limited problem awareness, which is also found in previous research (cf. De Los Reyes & Kazdin, 2005; Youngstrom, Loeber, & Stouthamer-Loeber, M., 2000). As we state in the limitations section, we unfortunately were not able to collect repeated measurements for parents, in addition to the self-reports of adolescents. Given the low rate of self-reported behavioral problems by adolescents, the reliance on adolescent self-report data might have compromised the validity and sensitivity of the repeated measurements.

Another finding contrasting with our expectations is that the development of adolescents’ problems over time is not moderated by treatment duration. This indicates that adolescents with a relatively short stay do not improve quicker than adolescents with a longer length of stay in SRC. We observe that adolescents with a longer stay (>6 months) continue to experience a decrease in internalizing and externalizing behavioral problems after being in care for over six months, ultimately leading to lower levels of these problems. These findings are in line with the outcomes educational residential care settings in Israel, showing adolescents with a longer stay to exhibit fewer emotional and behavioral problems (Hoffnung Assouline & Attar-Schwartz, 2020). Although it is Dutch policy that the duration of the placement of an adolescent in SRC is as short as possible, and as long as necessary, there is no prescribed maximum duration. Therefore, a placement duration of over six months is not necessarily in conflict with Dutch policy (Dutch Government, n.d.). However, organizations providing SRC are experiencing increasing pressure from society to further shorten the duration of SRC placements. And one may wonder whether there are cases when such a prolonged stay in SRC is warranted. Could intensive ambulant or residential care lead to the same outcome, when the need for secure care no longer exists? Our results, showing adolescents to keep developing in a positive way, also after six months of stay in SRC and beyond, seem to indicate that a relatively long stay can be helpful for some adolescents. However, as we shall see below, other adolescents with comparable levels of problem behavior do leave SRC after a significantly shorter period (i.e., three to six months).

Our findings also show that the change of the total, internalizing, and externalizing behavior problems, and attention problems over time varied widely between adolescents. We determined four types of development: a decrease of problems, an increase of problems, no change of problems over time and something we called a ‘U-shape’ development (i.e., at first a decrease of problems, followed by an increase of problems). Especially the ‘U-shape’ type of development calls for further examination, because, according to the level of their problem behavior, these adolescents might have been better off leaving SRC after a shorter period of time, more specifically at the time their problems were the least severe. Adolescents who are not discharged from SRC while performing at their best can easily lose motivation for treatment. Losing treatment motivation can be an explanation for their problem behavior to worsen (Van der Helm et al., 2014). Strikingly, other adolescents with a comparable level of behavioral problems do discharge from SRC after a significantly shorter period. Possibly this difference is caused by factors other than the (behavioral) problems of the adolescents. In an attempt to explain these differences, adolescents with a relatively short stay were compared with adolescents with a relatively long stay. As we can see from our findings, the destination after discharge of the adolescents offers no possible explanation, since it is not related to length of stay. However, waiting lists for follow-up care can pose a threat to shortening the length of stay in SRC. It is a serious possibility that for some of the adolescents follow-up care was not (yet) available at the ideal time of discharge from SRC. We know from practice that this sometimes leads to extending the treatment duration in SRC, which can have negative effects on the adolescents’ treatment motivation and behavioral problems. For example, a previous study in Dutch SRC has shown approximately 10%–20% of adolescents experience a prolonged stay in SRC due to the absence/availability of suitable follow-up care (Hospers & Van der Zwaan, 2018).

To explain the differing lengths of stay between adolescents we compared adolescents’ problems at admission for adolescents with a relatively short stay to adolescents with a long stay. We expected adolescents with a short stay to show less serious problems (i.e., total, internalizing and externalizing behavioral problems, attention problems, and family problems) at admission, compared to adolescents with a long stay. Remarkably, our findings did not meet our expectations. A possible explanation for the lack of differences found between adolescents with a relatively short stay compared to adolescents with a relatively long stay is that our study is performed within a relatively small sample. The small sample reduces the likelihood of detecting a significant difference between the two groups, increasing the chance of a type-II-error. We only found that adolescents remaining in care for a longer period are more often female. However, our findings indicate that adolescents’ problems at admission do not determine the length of stay. This contrasts findings by Dirkse et al. (2018) in Dutch SRC that the more risk factors present in the adolescent, the longer the SRC placement duration. Dirkse et al. explored 57 risk factors, divided over 11 domains: individual factors, substance abuse, daily activities, internalizing problem behavior, externalizing problem behavior, other problem behavior, presence of trauma, sexual abuse, sexual transgressive behavior, family, and the context. However, they did not investigate the individual contribution of the risk factors to adolescents’ length of stay. These findings suggest that length of stay is possibly not solely determined by behavioral problems, as we have measured, but also by (many) other risk factors. The different locations do not serve as a possible explanation as well, since the average length of stay of the adolescents in Hestia (202 days) and Midgaard (203 days) are similar. However, from a previous study within the populations of Hestia and Midgaard we know girls suffer from (severe) trauma problems more often than boys do (Gutterswijk et al., 2022). The presence of trauma problems can serve as a possible explanation for the statistically significant longer length of stay of girls in SRC than the length of stay of boys, since the treatment of trauma problems can take a very long time. Furthermore, we believe additional research is needed to identify possible explanations for the longer length of stay of girls in SRC.

Based on previous research (Blankestein et al., 2021), we also expected an association between the use of family therapy and a shorter SRC placement. However, this expectation was not confirmed by the results of our analyses. Furthermore, we expected adolescents with a long stay to transfer back home more often than adolescents with a short stay, since the transfer back home is expected to require more extensive preparation. It was assumed plausible that adolescents who progress to follow-up care are more suitable for this transfer after a shorter period of treatment in SRC, than adolescents who return home. In contrast to our expectations, we failed to find any differences in destination after discharge for adolescents staying in SRC for a short period of time, compared to adolescents staying in SRC for a long period of time. A possible explanation is that follow-up care is not always immediately available when adolescents are ready for the next step in their trajectory (Hammink et al., 2016; Hospers & Van der Zwaan, 2018; Van Dam et al., 2017). Another possible explanation is, again, that we failed to find a statistically significant difference between adolescents with a relatively short stay compared to adolescents with a relatively stay, because of the relatively small sample we used in this study.

We did however find that adolescents who stay in care for a longer period of time are, on average, older at admission. It is conceivable that older adolescents are progressing to living on their own after discharge more often than younger adolescents do, and that it takes more time to prepare an adolescent to live on its own than to return home or to progress to a less intensive type of care. However, analysis already showed that there are no differences in destination after discharge between adolescents with a short and long stay in SRC. In addition, this finding contrasts with the findings of Dirkse et al. (2018), who found younger adolescents to stay in care for a longer period of time. A possible explanation for our findings is that younger adolescents are more receptive to the help than older adolescents, which results in the former developing positively quicker.

Our last aim, as part of the second research question, was to compare follow-up scores (i.e., 3 months after discharge) for adolescents with a short stay compared to the scores of adolescents with a long stay. We expected that adolescents leaving SRC within six months would show more serious behavioral problems at follow-up than adolescents staying in care for over six months. Our findings showed that internalizing and externalizing behavioral problems, and for attention problem severity three months after discharge were the same for adolescents with a short stay and long stay. This seems to indicate that adolescents have left SRC at the right moment in time. Furthermore, it indicates that the problem scores of adolescents staying in SRC for less than six months are relatively stable over time after discharge or even improve and that adolescents staying in care for over six months slightly deteriorated after discharge. These findings are in contrast to the outcomes of family-style residential care in the USA, where adolescents staying in care for a longer period of time showed more positive outcomes regarding education, employment and delinquency at 24 month follow-up (Huefner et al., 2018). Although not part of this study, it would be interesting to see if the adolescents with a short stay in our sample indeed make repeated use of SRC more often than adolescents with a long stay, in line with findings by Koster et al. (2016). The level of their problems at follow-up does not make this plausible.

### Limitations

This study has several limitations. To maximize response rates questionnaires were provided face-to-face and not digitally. Collecting repeated measurements this way is extremely labor-intensive and continuously motivating adolescents in secure residential care to participate is hard, therefore, the sample size is relatively small, causing a threat to statistical power. It is possible that with a larger sample size more differences would have been found between adolescents with a short stay compared to adolescents with a long stay. Furthermore, only self-reports were used, while previous research showed parents to report their childrens’ problems to be more severe than adolescents reported their problems themselves. A possible explanation for youth reporting fewer problems is a lack of problem awareness. When problem awareness increases overtime, this reduces the chance of reporting a significant positive treatment effect. Unfortunately, research experiences in clinical practice taught us that conducting biweekly measurements among parents was not expected to yield an acceptable response rate (>25%), furthermore, since parents were asked to fill out several questionnaires at admission, discharge and follow-up, adding repeated measurements would have overburdened parents.

The repeated measurements contain a significant amount of missing values, also posing a threat to statistical power. Furthermore, missing data improves the risk of bias and decreases the generalizability of the data. We, however, have attempted to reduce these risks by means of multiple imputation techniques.

Fourth, we used an AB design without a baseline before treatment, because an ABA design (A = baseline before treatment, B = treatment in SRC and A = follow-up measurements after treatment) is not possible in SRC. To accommodate referral to SRC a judge must authorize placement. When the judge authorizes placement, the adolescent is directly admitted to an SRC facility, making it impossible to collect baseline measurement before treatment. Furthermore, we attempted to collect measurements after treatment, but this resulted in an insufficient number of responses. Comparing the treatment phase to a baseline before treatment may have led to other findings.

A fifth limitation is that we had no information about the decision to end the placement in SRC for the individual adolescents. This information could have provided an explanation for the treatment duration of SRC for individual adolescents.

To explore similarities or differences at time of follow-up, we used non-parametric tests. However, non-parametric tests are known to be more conservative and having less statistical power than parametric tests. Non-parametric tests are also more likely to produce a Type II error than parametric tests. Therefore, it could have been possible that there were indeed differences at follow-up we overlooked by using non-parametric tests.

### Implications for Practice and Future Research

We encourage researchers to do more in-depth research, for example by using mixed methods single case research (MMSCR) (i.e., by integrating quantitative and qualitative research methods) (Onghena et al., 2019), into reasons for terminating treatment in SRC, to explore possible explanations for the differing treatment duration of SRC between adolescents. This information can be a further step in determining the ideal length of stay for adolescents in SRC and possibilities to shorten the length of stay of adolescents in SRC. Furthermore, in line with our findings, we recommend intensive screening of adolescents’ problems and strengths at admission and during treatment by care professionals. Intensive screening can help practitioners to decide whether SRC is the most suitable type of care for an adolescent, and it helps to determine whether treatment is still effective after a certain period. Intensive screening at admission and over time is not yet common practice in SRC. Combined with face-to-face measurements, we recommend using a digital app to follow adolescents during treatment to capture real-time ecological treatment processes (cf. Altman et al., 2020; Jensen-Doss et al., 2020). Although more and more youth care organizations have adopted the method of data-driven decision making, future research can help gain knowledge about how to effectively implement this philosophy into every level of a youth care organization.

### Conclusion

Our findings show that most adolescents referred to treatment in SRC fail to benefit from this type of care. Only a specific group of adolescents improves regarding their behavioral problems. It is plausible that adolescents are referred to SRC when no alternative treatment is available. Intensive screening can prevent adolescents to be referred to SRC unjustified. Furthermore, alternative interventions for adolescents that fail to benefit from SRC are urgently needed.

Some adolescents continue to develop positively after a stay of over six months, however, the level of their individual and family problems show no justification for continuing their treatment for such a long time. Repeated screening is advised, since discharging from SRC seems possible when the most serious developmental threats have decreased. Consequently, this could help shortening the length of stay of adolescents in SRC. Further research is necessary to gain knowledge about the differences of length of stay of adolescents in SRC.

## Supplemental Material

Supplemental Material - Reducing Behavioral Problems and Treatment Duration of Adolescents in Secure Residential Care: A Multiple Single-Case Experimental Design Study Supplemental Material for Reducing Behavioral Problems and Treatment Duration of Adolescents in Secure Residential Care: A Multiple Single-Case Experimental Design Study by Raymond V. Gutterswijk, Chris H. Z. Kuiper, Annemiek T. Harder, Frank C. P. van der Horst, Bruno R. Bocanegra, and Peter Prinzie in Evaluation & the Health Professions.
