# Supplementary material for: Reducing Behavioral Problems and Treatment Duration of Adolescents in Secure Residential Care: A Multiple Single-Case Experimental Design Study
Source: Eval Health Prof. 2024 Dec 2;48(1):95–111. doi: 10.1177/01632787241228552 (PMC11909781; doi:10.1177/01632787241228552)
Supplement: Supplemental Material - Reducing Behavioral Problems and Treatment Duration of Adolescents in Secure Residential Care: A Multiple Single-Case Experimental Design Study [file sj-pdf-1-ehp-10.1177_01632787241228552.pdf]

Appendix A

Plots of behavioral development of the adolescents over time

Total behavioral problems

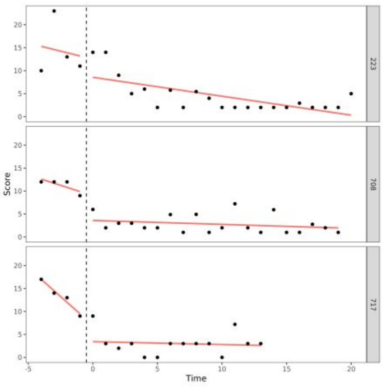

Plots of total behavioral problems of adolescents – decrease over time

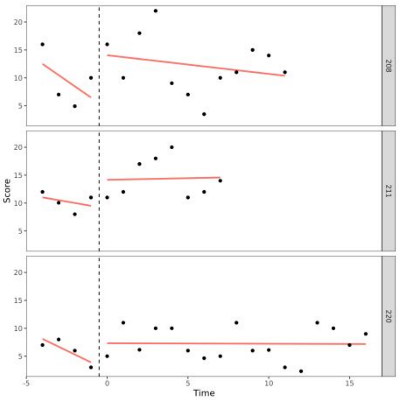

Plot of total behavioral problems of an adolescent – no change over time

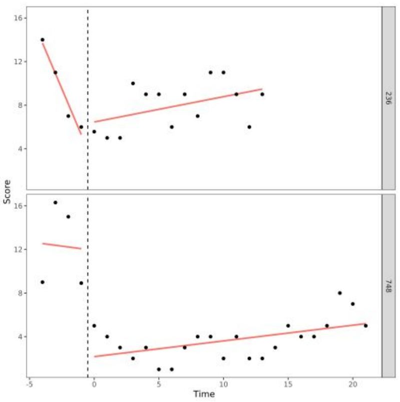

Plots of total behavioral problems of adolescents – decrease and increase over time (U-shape)

Internalizing behavioral problems

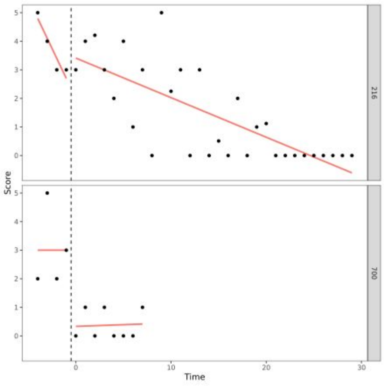

Plots of internalizing behavioral problems of adolescents – decrease over time

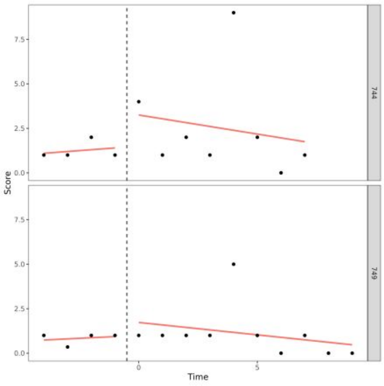

Plots of internalizing behavioral problems of adolescents – no change over time

Externalizing behavioral problems

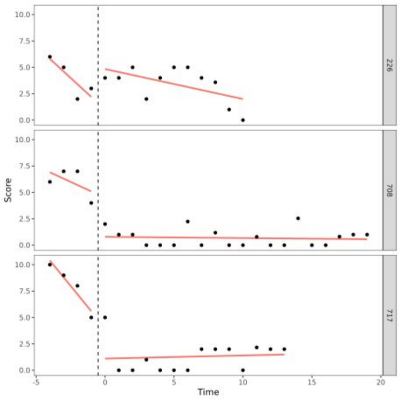

Plots of externalizing behavioral problems of adolescents – decrease over time

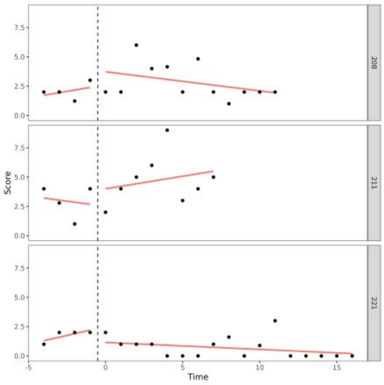

Plots of externalizing behavioral problems of adolescents – no change over time

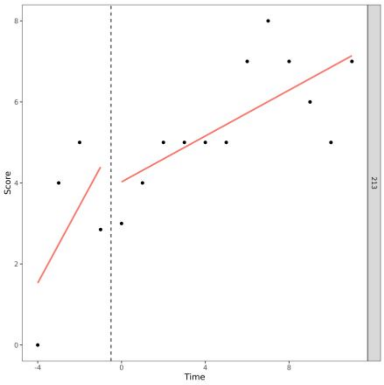

Plot of externalizing problem behavior of an adolescent – increase over time

Attention problems

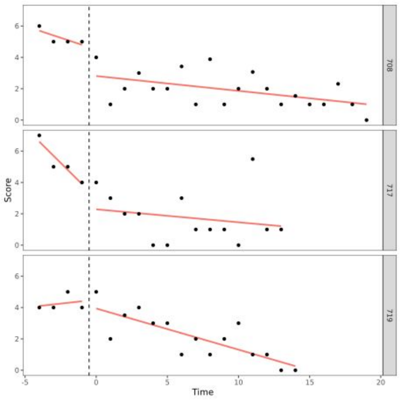

Plots of attention problems of adolescents – decrease over time

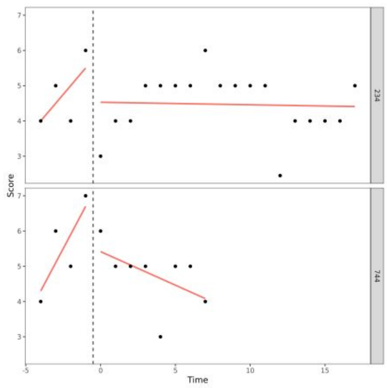

Plots of attention problems of adolescents – no change over time

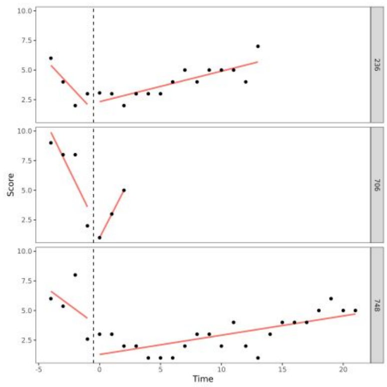

Plots of attention problems of adolescents – decrease and increase over time (U-shape)

Appendix B

Results of one-level analyses of behavioral development over time.

Total behavioral problems

Participant	*β1*	*β2*	*β3*
1	3.009*	-3.645	-3.305*
2	-2.008	9.588	1.674
3	0.389	-2.477	-0.541
4	-1	4.202	1.022
5	-0.505	5.167	0.565
6	-1.9	2.744	1.612
7	-1.607	1.907	2.555*
8	-0.5	-2.494	0.352
9	-0.2	0.237	0.02
10	-1.4	4.821	1.391
11	-0.2	-2.256	-0.095
12	-1	8.582	1.184
13	-0.7	-3.943	0.288
14	-0.3	4.335	-0.792
15	1.6	1.286	-1.915
16	-0.53	-3.688	0.132
17	0.7	-1.978	-0.776
18	-2.8	3.961	3.032**
19	-0.5	-2.822	0.233
20	-0.5	2.572	0.454
21	0.3	-2.083	-0.062
22	-0.7	0.489	0.133
23	-2*	-0.417	2.155*
24	1.3*	-3.2	-2.6**
25	2.06**	-4.265*	-4.06**
26	-3.19	2.453	1.193
27	-3.2	-0.833	5.7
28	-0.203	-4.697	0.382
29	-0.9	-5.397*	0.814
30	-1.1*	0.8	1.4
31	-2.5*	-3.582	2.438*
32	0	0.015	-0.396
33	-3.687*	12.604**	1.862
34	-1.3	0.778	1.284
35	1.2	-2.048	-0.944
36	-8.5*	18.1*	5.6
37	0.8	-0.667	-0.717
38	0.944	10.92	-8.544
39	-0.157	-9.74**	0.301
40	0.9	0.218	-1.082

* $p < .05$
**$p < .01$

Externalizing behavioral problems

Participant	*β1*	*β2*	*β3*
1	0.209	1.436	-0.304
2	0.223	1.113	-0.386
3	-0.184	0.453	0.137
4	0	-0.493	0.037
5	-0.179	1.5	0.393
6	-1.1	1.823	1.035
7	0.955	-1.324	-0.672
8	0.4	-1.443	-0.433
9	0.2	-0.712	-0.181
10	-0.2	0.1	0.228
11	0.3	-1.349	-0.359
12	-0.1	3.214	-0.329
13	-0.5	0.369	0.393
14	-1.2	3.837	0.916
15	0	1.017	-0.041
16	-1.264	2.807	1.254
17	0.2	-0.557	-0.259
18	-0.4	0.955	0.487
19	-0.7	1.124	0.58
20	-0.7	2.068	0.75
21	0.6	-2.5	-0.457
22	-0.4	0.356	0.283
23	-2.1*	2.667	0.517*
24	0.5	-2.1	-1
25	-0.7	1	0.2
26	-1582	2.515	1.187
27	-0.2	-0.167	0.2
28	-0.073	-0.773	0.386
29	-0.6	-3.709**	0.588
30	-0.3	0.9	0.2
31	-1.6*	-2.895	1.63*
32	-0.2	0.48	0.107
33	-0.766	3.163	0.049
34	-1.1**	1.684**	1.066**
35	0	0.738	-0.004
36	-0.8	3.5*	-0.2
37	-0.1	-0.333	0.588
38	-0.018	6.86	-3.182
39	0.303	-3.366**	-0.334
40	0.282	-0.627	-0.276

* $p < .05$
**$p < .01$

Internalizing behavioral problems

Participant	*β1*	*β2*	*β3*
1	-0.258	2.545	0.098
2	-2.021	6.861	1.744
3	0.184	0.12	-0.305
4	-1.2*	3.877*	1.154*
5	-0.337	2.333*	0.349
6	-0.6	-0.041	0.607
7	-0.843	-1.682	1.34
8	0	-1.436	-0.281
9	-0.7	1412	0.561
10	-1.9*	4.453	1.801
11	0	-0.254	-0.231
12	-0.4	4.886	0.851
13	-0.9	0.625	0.821
14	0.8	-0.38	-1.09*
15	0.4	1.289	-0.572
16	0.314	-3.032	-0.621
17	0	0.197	-0.008
18	-1.3	2.555	1.09
19	-0.2	-0.127	0.194
20	0	0.516	0.003
21	-0.3**	0.5	0.3*
22	-0.1	0.467	-0.183
23	0	-2667	0.012
24	0.5	-1	-1
25	0.083	1.593	-1.083
26	-1.52	1.889	0.309
27	-0.9	-0.167	1.4
28	-0.955	-1.719	1.033
29	0	0.205	0.016
30	-0.6	1	0.6
31	0	-0.112	0.039
32	0.1	-0.417	-0.093
33	-0.162	2.136	-0.207
34	0	0.145	0
35	0	-0.186	-0.017
36	-3.6**	4.9	3*
37	0.1	1.75	-0.314
38	-0.101	4.57	-1.999
39	0.118	-1.362*	-0.146
40	0.065	0.727	-0.204

* $p < .05$
**$p < .01$

Attention problems

Participant	*β1*	*β2*	*β3*
1	1.321*	-1.922	-1.361*
2	-0.16	2.711	0.226
3	-0.04	-0.76	-0.128
4	0.2	1.814	-0.267
5	-0.222	1.333	0.055
6	-0.2	0.918	-0.013
7	-0.073	0.557	0.265
8	-0.9	0.573	1.122
9	0.3	-0.235	-0.366
10	-0.8	2.529	0.776
11	-0.2	-1.175	0.2
12	-0.5	1.114	0.534
13	0.7	-4.824	-0.904
14	0.1	0.835	-0.592
15	1.2*	-0.839	-1.272
16	-1.032	2.718	1.143
17	0.5	-1.47	-0.507
18	-1.1*	1.332	1.357**
19	0.4	-3.622	-0.467
20	0.2	0.318	-0.318
21	0.3	0	-0.479
22	-0.2	-0.333	0.033
23	0.1	-0.417	-0.374
24	0.1	0.2	-0.2
25	0.708	-1.673	-1.208
26	-0.56	0.658	0.418
27	-2.1	-0.5	4.1
28	0.564	-2.764	-0.565
29	-0.3	-1.69	0.205
30	-0.2	-1.1	0.6
31	-0.9	-0.714	0.817
32	0.1	-0.561	-0.363
33	-1.601*	4.05*	1.06
34	-0.2	-0.789	0.194
35	1.2*	-1.761	-0.999
36	-2.3	6.7	1
37	0.8	-2083	-0.99*
38	0.172	2.46	-2.472
39	-0.759	-2298	0.922
40	0.296	0.118	-0.344

* $p < .05$
**$p < .01$
